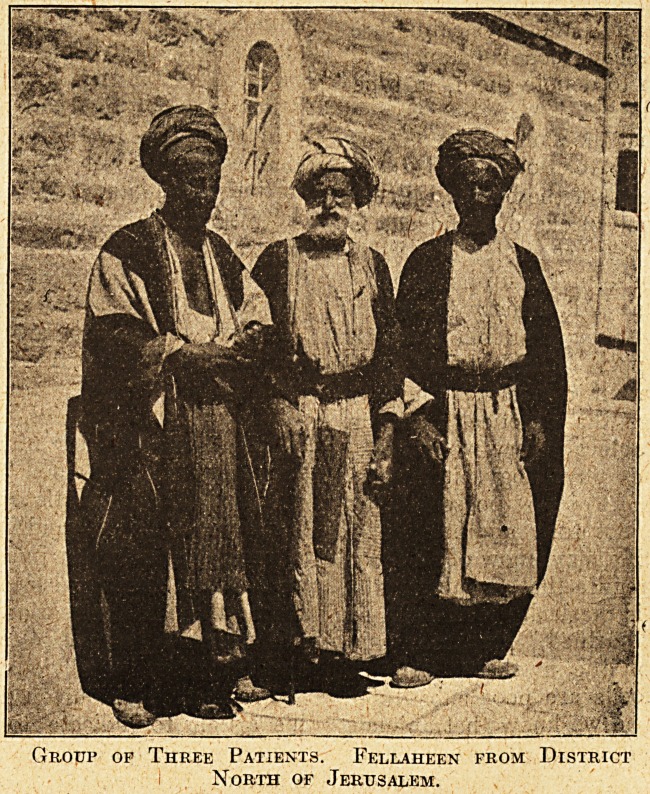# Hospital Life in Jerusalem

**Published:** 1919-06-21

**Authors:** 


### HOSPITAL LIFE IN JERUSALEM.

The history of British Hospitals in the East necessarily has a flavour not to be found in those with whose working we are all familiar. This is the case with the British Ophthalmic Hospital, Jerusalem, belonging to the Grand Priory of the Order of S. John of Jerusalem in England. English people have heard probably that the hospital continued its work till the end of the war, when the Turks converted it into an ammunition depot, and removed its equipment.

When Jerusalem was on the point of surrender, the Turks blew it up just as they were leaving. The outer walls remain, but the hospital was largely destroyed, though sufficient repairs have been carried out to enable it to be re-opened for out-patients. Its commanding position along the Valley of Hinnom fits an institution which was unique in Palestine, and the sole scientific hope of the large proportion of those afflicted with diseases of the eye.

GROUP OF THREE PATIENTS. FELLAHEEN FROM DISTRICT NORTH OF JERUSALEM.

The Order, the first of the Orders of Christian knighthood, was of course established for the recovery of the Holy Sepulchre in the eleventh century. But Jerusalem is said to have possessed its first hospital as early as the third century. In 1099, when Jerusalem was captured in the first crusade, the Hospice for Pilgrims, established by Charlemagne about 800 and destroyed in 1010, was restored. It was the renewal of hostilities which led the community, which many of the Crusaders joined, to divert itself from a body of healers to a military organisation. In 1187, when the Christians were driven from Jerusalem by Saladin, the knights retreated first to Acre then to Rhodes (1310), and then (1522) to Malta, which they fortified till in 1798 they capitulated to Napoleon.

It was not, however, till 1882 that the members of the English branch of the Order decided in the Jerusalem Chamber at Westminster Abbey "to establish a British Hospice and Ophthalmic Dispensary at Jerusalem." Dr. W. J. Waddell began work there on December 4, 1882. It was a wish of the English branch to establish in Jerusalem, as other nations had done, a charity unconnected with proselytising.

For the sake of this quality the Turks supported it, and the Sultan authorised the Order to open a hospital, and even gave a site and permission to purchase further land. The building was a Turkish house of stone, on the Bethlehem road, with eighteen rooms, to which additions were made. The history of the hospital records that in 1911 Sir Charles Weston, after a visit, advised the erection of a laboratory, which was since built. The staff before the war consisted of two resident surgeons, a matron, and a nurse. The first permanent assistant surgeon was appointed in 1903. It is noteworthy that the post of matron was often filled by the wives of the surgeons. There is a native staff.

The annual report for 1913 showed that 1,262 in-patients, and 9,604 out-patients had been treated, while 2,542 operations had been performed. The work was much appreciated by the Turks and foreign officials, and in 1888 the Orthodox Greek Patriarch made an annual grant to maintain a bed, and the Anglican Bishop endowed one.

## Figures and Tables

**Figure f1:**